# RNA profile diversity across arthropoda: guidelines, methodological artifacts, and expected outcomes

**DOI:** 10.1093/biomethods/bpy012

**Published:** 2018-12-15

**Authors:** Danielle M DeLeo, Jorge L Pérez-Moreno, Hernán Vázquez-Miranda, Heather D Bracken-Grissom

**Affiliations:** Department of Biological Sciences, Florida International University-Biscayne Bay Campus, North Miami, FL, USA

**Keywords:** gap deletion, denaturation, RNAseq, nucleic acids, transcriptomics, genomics, crustaceans, insects, invertebrates

## Abstract

High-quality RNA is an important precursor for high-throughput RNA sequencing (RNAseq) and subsequent analyses. However, the primary metric used to assess RNA quality, the RNA Integrity Number (RIN), was developed based on model bacterial and vertebrate organisms. Though the phenomenon is not widely recognized, invertebrate 28S ribosomal RNA (rRNA) is highly prone to a form of denaturation known as gap deletion, in which the subunit collapses into two smaller fragments. In many nonmodel invertebrates, this collapse of the 28S subunit appears as a single band similar in size to the 18S rRNA subunit. This phenomenon is hypothesized to be commonplace among arthropods and is often misinterpreted as a “degraded” rRNA profile. The limited characterization of gap deletion in arthropods, a highly diverse group, as well as other nonmodel invertebrates, often biases RNA quality assessments. To test whether the collapse of 28S is a general pattern or a methodological artifact, we sampled more than half of the major lineages within Arthropoda. We found that the 28S collapse is present in ∼90% of the species sampled. Nevertheless, RNA profiles exhibit considerable diversity with a range of banding patterns. High-throughput RNAseq and subsequent assembly of high-quality transcriptomes from select arthropod species exhibiting collapsed 28S subunits further illustrates the limitations of current RIN proxies in accurately characterizing RNA quality in nonmodel organisms. Furthermore, we show that this form of 28S denaturation, which is often mistaken for true “degradation,” can occur at relatively low temperatures.

## Introduction

Next-generation RNA sequencing (RNAseq) has become a revolutionary tool for RNA-based characterization studies. Similar to traditional quantification methods (i.e., quantitative polymerase chain reaction (PCR)), it can now be performed in most modern laboratories with relative ease and accuracy [1] while yielding more precise and robust datasets [2]. For this reason, RNAseq has become a powerful technique for studying an array of biological questions in both model and nonmodel organisms, including *de novo* transcriptome reconstruction and profiling, gene discovery and functional analyses, differential gene expression studies [3], and evolutionary investigations (e.g., [4]). Moreover, continued advancements to sequencing technologies and analysis software have expanded the reach and utility of RNAseq to include microscopic inputs (singe-cell RNAseq), enabling spatial mapping and tissue reconstruction [5, 6].

Compared with DNA, total RNA extraction and sequencing poses nontrivial methodological challenges to maintain RNA stability. Due to its ephemeral nature, RNA is prone to cellular degradation [7] from the initial cellular lysis step to final visualization and quantitation. In addition, ribonucleases (RNases), ubiquitous enzymes present in cells and the environment can further degrade RNA if proper care is not taken during sample preservation, extraction, and downstream protocols [7]. To prevent degradation and loss of transcriptomic information, most extraction guidelines suggest using dedicated equipment in isolated areas and frequent, thorough RNase decontamination throughout the protocol. Ideally, samples should be flash frozen or preserved in specialized salt-saturated buffers (e.g., *RNAlater*^®^; [8]), when the use of fresh tissue is not feasible, and kept frozen at −80°C prior to extraction and post-isolation to prevent native degradation. These rigorous methodological demands portray the labile nature of RNA.

Historically, RNA was assumed to be of high quality if the ratio of large to small ribosomal subunits (28S:18S) was ∼2 or higher [9], though this method of assessment is sample consuming and imprecise [10]. Presently, there are two common methods used to test for RNA integrity and degradation after extraction: (i) visualization via denaturing (agarose) gel electrophoresis and (ii) fragment size chip electropherograms on a Bioanalyzer (Agilent). In particular, the Bioanalyzer step calculates the RNA integrity number (RIN) [11], now considered as the gold standard for successful library preparation and sequencing [10, 12–15]. The RIN model is estimated from RNA fragment distribution peaks and areas under the curve, and ranges from 1 to 10 (low to pristine quality, respectively). Sample RIN values of ≥7 are typically deemed as “high-quality” and appropriate for RNAseq (e.g., [16]), and often used as a cutoff for which sequencing facilities will guarantee results. Another common standard used to assess RNA integrity is ribosomal subunit size. Using rodent and human models, size has been estimated to be between 4400 and 5000 base pairs (bps) in the large 28S subunit and ∼2000 bp in the small 18S subunit [17]. Since RNA degradation can occur at any step in most protocols, it is paramount to determine appropriate reference standards and metrics for accurate RNA quality control [12].

Although relevant ribosomal RNA (rRNA) profile standards exist for several model organisms [18], most branches across the “Tree of Life” lack genomic references and resources. This paucity of genomic data is particularly visible across Arthropoda, a globally distributed group that encompasses >80% of animal diversity [19] and occupies a variety of niches from the poles to the tropics, high mountains, and deep-sea trenches. They fulfill significant roles in ecosystems (e.g., pollination and pests) and are of medical (i.e., disease vectors) and economic importance (e.g., fisheries) [20]. Due to their global relevance, the need for increased genomic references and reliable metrics to evaluate nucleic acid quality is crucial for the understanding of multiple evolutionary and ecological phenomena.

There are few recent studies that have evaluated the universality and suitability of “standard” RNA quality metrics for nonmodel organisms (e.g., [21]). Early observations of RNA profiles in insects documented a “hidden break” of the 28S subunit, also referred to as gap deletion, in which the 28S denatures and collapses (decouples) into two smaller fragments (α and β) [22, 23] and a short, variable stretch of rRNA is removed or deleted [24, 25). This produces an apparent “degraded” RNA profile where both 18S and 28S overlap in a single band. As RNAseq has become a common method for RNA investigations in recent years, more and more invertebrate rRNA profiles suggest that this “hidden break” – a form of 28S denaturation – is the norm rather than the exception [21, 26, 27].

Hidden breaks in RNA are correlated with the presence of a cleavage site characterized as an Uracil (U) and Adenine (A) or UAAU-rich segment in the rRNA loop [28]. High temperature exposure is believed to result in UAAU decoupling and the subsequent dissociation (collapse) of the 28S [21–23]. This dissociation was hypothesized to occur as Hydrogen-bonds in this AU-rich region melt – the strength of the bonds are comparable to the strength of the chemical changes associated with denaturing conditions [22]; the double bonds of the AU pairs are weaker relative to the triple bonds of Guanine-Cytosine (GC) associations [29]. Differences in base-stacking stability [30], secondary structures and enzyme-dependent cleavage events are also thought to play a role [25]. Likewise, mutations in this UAAU-rich segment are thought to prevent this dissociation [28]. In a recent study, McCarthy *et al.* [21] hypothesized that exposing RNA to heat at 70°C, mainly for electropherogram preparation in a Bioanalyzer, was responsible for the 28S subunit collapse. However, there are additional steps often used in RNA extraction protocols, such as gel electrophoresis and deoxyribonuclease (DNase) applications for DNA removal, which may also expose samples to temperatures high enough to trigger 28S cleavage. As DNase treatments can be performed at relatively low temperatures (∼37°C) for enzymes that do not require deactivation at 70°C, and temperature can be regulated during electrophoresis (e.g., ice bath and chilled buffers), 70°C may be an exaggerated upper limit for this denaturation to occur. Given the UAAU loop region is sensitive to heat, DNase enzyme activation temperatures of 37°C may result in 28S denaturation prior to 70°C exposure, and possibly result in different “degraded” profiles. Moreover, wider taxon sampling may reveal variable processing of this gap (loop) region, including insertions/deletions of additional UAAU-rich segments, yielding additional RNA profiles.

Here we document the range of variation among RNA extraction profiles for the major lineages within Arthropoda, with dense taxonomic sampling. Our primary motivation is to disentangle natural patterns of subunit denaturing from true degradation and methodological artifacts. Data available from prior studies are a composite of multiple extraction protocols from different laboratories that can further obscure these artifacts. This study was done using standardized reagents and conditions to improve comparisons among taxa. High-throughput RNAseq and *de novo* transcriptome assembly for two representative arthropod species exhibiting seemingly denatured RNA profiles provide further support for high-quality RNA despite evidence of 28S subunit collapse. Findings from this effort will aid future nucleic acid quality assessments for arthropods and other nonmodel invertebrates and will provide methodological insight for laboratories beginning to conduct RNA-based research.

## Materials and methods

### Arthropod lineages

We selected representative lineages covering nine classes across the four extant Arthropod subphyla [31, 32] (Table 1, *n* = total number of individuals sampled within each subphyla): Chelicerata (*n* = 4), Myriapoda (*n* = 11), Crustacea (*n* = 24), and Hexapoda (*n* = 20). Within Chelicerata, we sampled Arachnida (e.g., spiders and scorpions) and Merostomata (horseshoe crabs); within Myriapoda, we sampled Chilopoda (centipedes) and Diplopoda (millipedes); within Crustacea, we sampled Branchiopoda (water fleas), Oligostraca (e.g., barnacles), Multicrustacea (e.g., copepods), and Malacostraca (e.g., crabs, shrimp, lobsters, isopods, and amphipods); and within Hexapoda, we sampled Insecta (insects) and Entognatha (springtails). Our lineage selection encompasses all current Arthropod subphyla, approximately two-thirds of the classes in both Chelicerata and Crustacea, all classes within Hexapoda and half of all classes in Myriapoda. To account for the large diversity within Hexapoda and Crustacea (“Pancrustacea,” [33]), we sampled some groups more heavily; approximately half of all orders in Insecta [34] and infraorders in Decapoda [35]. We were not able to sample Pycnogonida, Pauropoda, Symphyla, Remipedia, Ostracoda, Ichthyostraca, and Cephalocarida due to lack of available RNA quality tissue. Onychophora and Tardigrada are panarthropods considered as sister to Arthropoda, but their rarity and/or small size made their inclusion in this study unfeasible.

**Table 1:** Samples utilized in this study

Fig. 1	HBG	SubPhylum	Class	Order	Species	Common name	Tissue	RNA bands
A01	4858	Crustacea	Malacostraca	Isopoda	*Caecidotea cf. communis*	Isopod	Whole	2^b^
E07	2867	Crustacea	Malacostraca	Isopoda	*Asellus aquaticus*	River isopod 1	Whole	3
E08	2873	Crustacea	Malacostraca	Isopoda	*Asellus aquaticus*	River isopod 2	Whole	3
E09	2861-2	Crustacea	Malacostraca	Isopoda	*Asellus aquaticus*	River isopod 3	Whole (2x)	3
F05	5062	Crustacea	Malacostraca	Isopoda	*Cubaris cf. murinus*	Land isopod 1	Whole	5^Δ^
F06	5063	Crustacea	Malacostraca	Isopoda	*Cubaris cf. murinus*	Land isopod 2	Whole	5
F07	5064	Crustacea	Malacostraca	Isopoda	*Cubaris cf. murinus*	Land isopod 3	Whole	5
A09	4866	Crustacea	Malacostraca	Amphipoda	*Gammarus pulex*	Amphipod	Whole	1
E05	2850	Crustacea	Malacostraca	Amphipoda	*Niphargus hrabei*	River Amphipod 2	Whole	1
E06	2855	Crustacea	Malacostraca	Amphipoda	*Niphargus hrabei*	River Amphipod 3	Whole	1
B11	2843-4	Crustacea	Malacostraca	Amphipoda	*Niphargus hrabei*	River amphipod 1	Whole (2x)	1
B02	4870	Crustacea	Malacostraca	Decapoda	*Stenopus hispidus*	Coral-banded Shrimp	1 chelae	2^b^
E02	3047	Crustacea	Malacostraca	Decapoda	*Oplophorus spinosus*	Pelagic shrimp	Eyes	2^b^
E04	3180	Crustacea	Malacostraca	Decapoda	*Farfantepenneaeus duorarum*	Pink shrimp	1 eye	1
D02	4893	Crustacea	Malacostraca	Decapoda	*Clibanarius tricolor*	Three-colored hermit crab	All legs	4
E03	3162	Crustacea	Malacostraca	Decapoda	*Eurypanopeus depressus*	Mud crab	Gill	2
F08	5273	Crustacea	Malacostraca	Decapoda	*Calinectes sapidus*	Blue Crab	One claw	3
F09	3018.E	Crustacea	Malacostraca	Decapoda	*Barbouria cubensis*	Cave shrimp 1	Eyes	2
F10	3018.A	Crustacea	Malacostraca	Decapoda	*Barbouria cubensis*	Cave shrimp 2	Antennae	2
F11	3018.S	Crustacea	Malacostraca	Decapoda	*Barbouria cubensis*	Cave shrimp 3	SDO	2
B08	4876	Crustacea	Malacostraca	Holocarida	*Gonodactylus sp*	Mantis shrimp	Cephalothorax	1
C06	4886	Crustacea	Branchiopoda	Cladocera	*Daphnia magna*	Water flea	Whole (10x)	3
B09	4878	Crustacea	Copepoda^a^	Calanoida	*Cyclops sp*	Freshwater copepod	Whole	2^b^
C05	4885	Crustacea	Thecostraca^a^	Sessilia	*Amphibalanus eburneus*	Ivory barnacle	Whole (4x)	1
A02	4859	Chelicerata	Arachnida	Araneae	*Nephila clavipes *	Golden web Orb spider	Head	1
B04	4872	Chelicerata	Arachnida	Scorpiones	*Hoffmannius spinigerus*	Stripe-tailed scorpion	1 leg	1
D05	4898	Chelicerata	Arachnida	Araneae	*Gasteracantha cancriformis*	Crab Spider	Head	1
B05	4873	Chelicerata	Xiphosura	Xiphosurida	*Limulus polyphemus*	Atlantic horseshoe crab	1 leg	2^b^
A03	4860	Hexapoda	Insecta	Thysanura	*Lepisma saccharina*	Silverfish 1	Whole	1
B06	4874	Hexapoda	Insecta	Thysanura	*Lepisma saccharina*	Silverfish 2	Whole	1
A04	4861	Hexapoda	Insecta	Diptera	*Drosophila melanogaster*	Fruitfly	Whole (10x)	1
C01	4880	Hexapoda	Insecta	Diptera	*Tipula sayi*	Crane fly	Whole	2^b^
A06	4863	Hexapoda	Insecta	Hymenoptera	*Apis mellifera*	Bee	Head	1
A10	4867	Hexapoda	Insecta	Hymenoptera	*Camponatus floridanus*	Florida carpenter ant	Two heads	1
A07	4864	Hexapoda	Insecta	Lepidoptera	*Ascia monuste*	Great Southern White	Head + abdomen	1
A08	4865	Hexapoda	Insecta	Coleoptera	*Tenebrio molitor*	Mealworm	head	1
C04	4884	Hexapoda	Insecta	Coleoptera	*Ignelater havaniensis*	Glowing click beetle	All photo phores	1
A11	4868	Hexapoda	Insecta	Orthoptera	*Acheta domestica*	Brown cricket	Head	1
B07	4875	Hexapoda	Insecta	Ephemeroptera	*Callibaetis floridianus*	Mayfly	Whole	1
B10	4879	Hexapoda	Insecta	Blattodea	*Blattella germanica*	German cockroach	Head	2^b^
C02	4881	Hexapoda	Insecta	Blattodea	*Reticulitermes flavipes*	Subterranean termite	Whole	2
D01	4892	Hexapoda	Insecta	Blattodea	*Periplaneta americana*	American cockroach	Head	1
D03	4894	Hexapoda	Insecta	Odonata	*Brachymesia gravida*	Four-spotted Pennant	Head	D
D04	4895	Hexapoda	Insecta	Odonata	*Brachymesia gravida*	Four-spotted Pennant	Head	2^b^
C03	4883	Hexapoda	Insecta	Mantodea	*Sphodromantis viridis*	African giant mantis	1 leg	1
C07	4887	Hexapoda	Insecta	Mecoptera	*Panorpa debilis*	Scorpion fly1 small	Whole	1
C08	4888	Hexapoda	Insecta	Mecoptera	*Panorpa debilis*	Scorpion fly2 big	Whole	1
B03	4871	Hexapoda	Entognata	Collembola	*Folsomia candida*	Giant springtail	Whole (20x)	1
A05	4862	Myriapoda	Diplopoda	Spirobolida	*Anadenobolus monilicornis*	Bumble bee millipede 1	Head+1/3 body	2
D06	5053	Myriapoda	Diplopoda	Spirobolida	*Anadenobolus monilicornis*	Bumble bee millipede 2	Head	2^Δ^
D07	5054	Myriapoda	Diplopoda	Spirobolida	*Anadenobolus monilicornis*	Bumble bee millipede 3	Head	2^Δ^
D08	5055	Myriapoda	Diplopoda	Spirobolida	*Anadenobolus monilicornis*	Bumble bee millipede 4	Head	2^Δ^
B01	4869	Myriapoda	Chilopoda	Scolopendromorpha	*Scolopendra polymorpha*	Tiger centipede	Head	4
F01	5058	Myriapoda	Chilopoda	Scolopendromorpha	*Hemiscolopendra marginata*	Blue centipede 1	Head	1
F02	5059	Myriapoda	Chilopoda	Scolopendromorpha	*Hemiscolopendra marginata*	Blue centipede 2	Head	1
F03	5060	Myriapoda	Chilopoda	Scolopendromorpha	*Hemiscolopendra marginata*	Blue centipede 3	Head	1
F04	5061	Myriapoda	Chilopoda	Scolopendromorpha	*Hemiscolopendra marginata*	Blue centipede 4	Head	1
E01	REF	Proteo- bacteria	Gamma- proteobacteria	Enterobacteriales	*Escherichia coli*	Qubit S2	1 μL	2^Δ^

**Figure 1:**
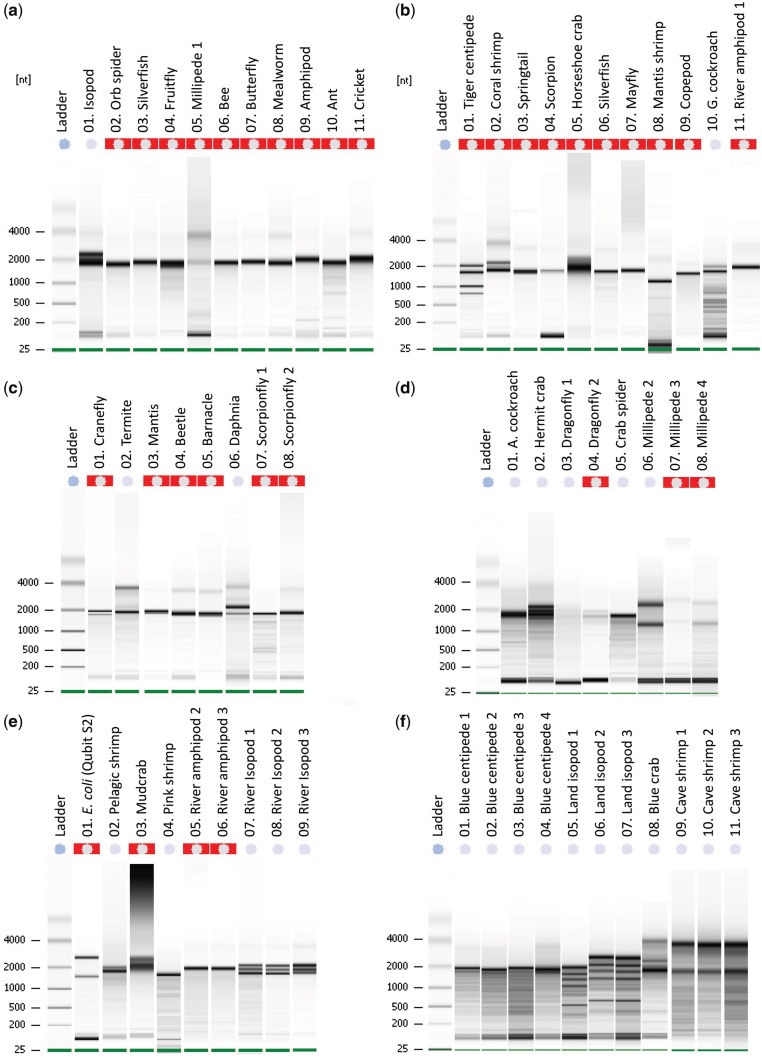
Bioanalyzer electropherograms of total RNA for a diverse array of Arthropod lineages. Sample information including taxonomic classifications are given in Table 1. Samples in Panels a–f are randomized (nontaxonomic order) but corresponding lane information is in Table 1. Ladders correspond to the picochip RNA size standard. Red (or highlighted) cells above a lane indicate that the RIN could not be calculated.

The first column corresponds to lanes on electropherograms (Fig. 1). HBG corresponds to voucher numbers in the Zoological collection at FIU. Subphylum to Species columns correspond to taxonomic classification (^a^Subclass). Common name stands for labels on Fig. 1. Tissue column indicates what part of the body was used for extraction and whether multiple individuals were pooled together. RNA bands column indicates the number of ribosomal fragments detected in Fig. 1.

D denotes a degraded sample, whereas Δ marks samples with shifted RNA bands (which would require manual adjustment for proper size estimation) and ^b^denotes samples with denatured profiles despite a two-banded pattern.

### Specimen preservation and tissue harvesting

We collected live specimens from the Biscayne Bay Campus of Florida International University (FIU) in North Miami, FL, USA, and brought them back to the lab for preservation. Some specimens were ordered from a commercial vendor (Carolina Biology, NC, USA) or donated by colleagues (see Acknowledgements section). Smaller individuals (bee size and below) were flash frozen and preserved at −80°C. Larger individuals were placed in *RNAlater* (Life Technologies, Foster City, CA, USA) with an abdominal incision and then placed at −80°C until RNA extraction. All specimens/or tissues are cataloged and deposited as vouchers (specimen or tissue) in the Florida International Crustacean Collection at FIU (Table 1).

### RNA extraction

We extracted RNA from 40 species across the four Arthropod subphyla. RNA was extracted from replicates where possible, including: the isopods *Asellus aquaticus* (*n* = 3), *Cubaris cf. murinus* (*n* = 3), amphipod *Niphargus hrabei* (*n* = 3), cave shrimp *Barbouria cubensis* (*n* = 3), silverfish *Lepisma saccharina* (*n* = 2), the insects *Brachymesia gravida* (*n* = 2) and *Panorpa debilis* (*n* = 2), the millipede *Anadenobolus monilicornis* (*n* = 4), and centipede *Hemiscolopendra marginata* (*n* = 4). Though RNA quality tissue replicates were difficult to obtain for some species, all species were included in the study regardless as an effort to more thoroughly survey RNA profile diversity across Arthropoda.

All stages of our RNA extraction protocol were carried out under a fume hood in a dedicated RNase-free room. Bleach (2% solution in distilled water) was applied to all surfaces, equipment, and reagent containers to remove RNases and secondarily sterilized with RNaseZAP^®^ (Life Technologies, Carlsbad, CA, USA). We isolated total RNA from varied tissues or body parts depending on the specimen size (see Table 1). For very small specimens, we used the entire body or pooled multiple individuals. Tissues were homogenized in 1 mL TRIZOL^®^ Reagent (Life Technologies, Carlsbad, CA, USA). Approximately 10–12 sterile 2 mm zirconia/ceramic beads were used in a MiniBead Beater (BioSpec, Bartlesville, OK, USA) for homogenization in a 2 mL screwcap tube, followed by the addition of chloroform (0.2 mL). Samples were mixed (shaken) vigorously by hand for 15  s and incubated at room temperature for 2–3 min. Phase separation was carried out by centrifuging samples (12 000*g* for 15 min) at 4°C, following manufacturer’s instructions, and carefully transferring only the top (clear) aqueous phase containing the RNA into new vials. RNA was then precipitated from the clear aqueous phase by mixing in (∼100%) isopropyl alcohol (0.5 mL) and glycogen (0.1 μL). Samples were then incubated at room temperature for 10 min and centrifuged (12 000*g* for 15 min) at 4°C. The supernatant was discarded, the RNA pellet washed twice with freshly made 75% ethanol (EtOH) and the supernatant was again discarded; 1 mL EtOH was added before centrifugation at 7500*g* for 5 min at 4°C (X2). Before elution, precipitated RNA was treated with DNases (Clonetech, Mountain View, CA, USA) to remove genomic DNA (15-min incubation at 37°C) following manufacturer’s guidelines. After repeating the isopropyl precipitation and ethanol washes (2×), total RNA was eluted in 50 μL of RNase-free water (Sigma, St Louis, MO, USA) and stored at −80°C.

RNA integrity was determined initially on a 1% agarose gel (made with Tris base, acetic acid and Edetic acid (EDTA) or TAE) treated with a 1% bleach solution and ran in cold buffer at 4°C to prevent RNA degradation during electrophoresis (e.g., 500 μL bleach diluted in 50 mL agarose TAE solution [36]). In order to determine the size of the 18S and 28S rRNA subunits and check for degraded samples, we added a RiboRuler RNA ladder (ThermoFisher, USA) to the agarose gel. The RNA concentration of each extraction was measured using a Qubit 2.0 fluorometer (Life Technologies, USA). Finally, we diluted a subsample from every extraction to 5 ng/μL and determined the RNA profile by measuring ribosomal number, size, and RIN number using a BioAnalyzer 2100 with RNA 6000 picochips (Agilent, USA) in the DNA Core Facility at FIU; all samples were processed without the heat exposure (70°C) typical of bioanalyzer protocols. Gloves were changed between each protocol step and RNaseZap was used liberally.

### High-throughput sequencing of RNA exhibiting 28S denaturation

RNA aliquots of the isopod *A. aquaticus* and amphipod *N. hrabei*, each exhibiting denatured RNA profiles indicative of 28S collapse (Fig. 3a and b, respectively), underwent mRNA isolation and complementary DNA (cDNA) library prep using the NEBNext^®^ Ultra^TM^ II Directional RNA Library Prep Kit for Illumina^®^. Libraries were then sequenced on an Illumina HiSeq4000 (for more details, see [37]). These data are available as part of the BioProject PRJNA476149. Raw data were quality checked, trimmed and assembled *de novo* according to the methods described in Pérez-Moreno *et al.* [37]. Briefly, the quality of raw sequencing data was assessed with FastQC [38] and trimmed with Trimmomatic v0.36 [39] using the following parameters: ILLUMINACLIP: 2: 30: 10 CROP: 140 HEADCROP: 20 LEADING: 15 TRAILING: 15 SLIDINGWINDOW: 4: 20 MINLEN: 36. High-quality adaptor trimmed data were assembled with Trinity v2.5.0 (minimum transcript length 200 bp; *k*-mer size 23). The resulting *de novo* transcriptome assembly was assessed using Transrate v1.0.3 [40] and Benchmarking Universal Single-Copy Orthologs (BUSCO) v3.0.2 [41] using OrthoDB’s Arthropod database of orthologous groups (*n* = 1066) [42] to determine assembly quality and completeness.

### Distinguishing true RNA degradation from analytical artifacts

High temperature exposure is considered as one of the determining factors behind the 28S subunit collapse [21, 22]. To test whether denaturation could occur at temperatures <70°C, we chose three representative lineages with differing rRNA profiles: millipede, centipede, and crab. We used a bacterial reference (total) RNA (*Escherichia coli*, Life Technologies, USA) as a noncleaved 28S positive control. RNA aliquots were taken from the three samples prior to the DNase treatment, precipitated, eluted, and kept at 4°C. We prepared a heat treatment gradient as recommended by the Bioanalyzer manufacturer, heating all samples in a MJ Research PTC-200 thermocycler, incubating for 2 min at a designated temperature using a nonheated lid, prior to cooling at 4°C. RNA from the four samples (including the positive control) were aliquoted into eight different temperature treatment sets (0.2 mL PCR strip tubes). Each set was placed across a temperature gradient ranging from 20°C to 90°C, at increments of 10°C. This range encompasses “room temperature” and standard DNase treatment temperatures, as well as temperatures 20°C higher than the recommended denaturing step in an attempt to force denaturation for comparison. After the eight tube sets were incubated at their corresponding temperature on the thermal gradient, we kept the samples at 4°C and stored them permanently at −80°C until they were run on both a 1% TAE bleach gel in cool buffer and Bioanalyzer picochips as described previously.

## Results

### An array of RNA profile patterns within arthropoda

In total, we found six distinct patterns of 28S denaturation (Table 1), and banding patterns were consistent across replicates. In most arthropod lineages, we found a consistent “denatured” or “collapsed” RNA profile, where the 28S subunit is denatured into equally sized α-β fragments (Fig. 1). This dominant pattern was found in 22 species and appeared on the gel as a single thick band resulting from the collapse of 28S and subsequent overlap with 18S at around 1900 bp (Fig. 1a–f); a size estimate reported previously for the honey bee *Apis mellifera* (27). We detected two prominent bands in the fruit fly, cranefly (Diptera), pelagic shrimp, banded-coral shrimp, blue crab, and mudcrab (Decapoda), at ∼2000 and 1900 bp, respectively. Three well-defined bands were detected in river isopods (Isopoda), marine crabs (Decapoda), and the water flea (Cladocera) at ∼2200, 2000, and 1900 bp. The hermit crab (Decapoda) appeared to have four bands of similar size between 1800 and 2200 bp although it is possible that the presence of minor degradation may have confounded this profile. The only lineage with four distinct bands was the tiger centipede (Chilopoda) at ∼2000, 1800, 1000 and 800 bp. Land isopods (Isopoda) showed five distinct bands at ∼2200, 2000, 1900, 1700 and 600 bp though some degradation was present. Lastly, we found a nondenatured, two-band profile for cave shrimp (Decapoda) and bumblebee millipede (Diplopoda) – with subunit bands at ∼3900 bp and 1900 bp, similar to the model rRNA references [17] (Fig. 1). It is important to note that the rRNA fragments of some replicate lanes appear to have shifted on the picochip (e.g., Fig. 1f, lane 05) and would require manual adjustments for accurate size estimation (see Table 1), though the banding patterns remain consistent.

Some samples did show “true” degradation <1800 bp including the hermit crab *Clibanarius tricolor* and one replicate of the dragonfly *Brachymesia gravida* (Table 1 and Fig. 1), which appeared as multiple bands on the Bioanalyzer traces. Likewise, residual DNA contamination was evident in the mud crab *Eurypanopeus depressus* after the DNase treatment (Fig. 1e–l); it is possible that the DNase was not as effective because a higher volume of tissue was available and used for this particular extraction. Many samples also exhibited remnants of the original size of the 28S subunit below the 4000 bp mark, appearing as a faint band and a shorter peak on the electropherograms (Fig. 1). Mean sizes for the original 28S and the 18S peaks suggest that, in arthropods, RNA profiles with no denaturation can be measured at ∼3900 bp and 1900 bp, respectively. Several samples showed multiple faint bands beyond 1900 bp, which may imply some level of degradation and/or remnants of extracted 5/5.8S ribosomal subunits and tRNAs (Fig. 1). 

### Ribosomal 28S denaturation can occur at “low” temperatures

**Figure 2:**
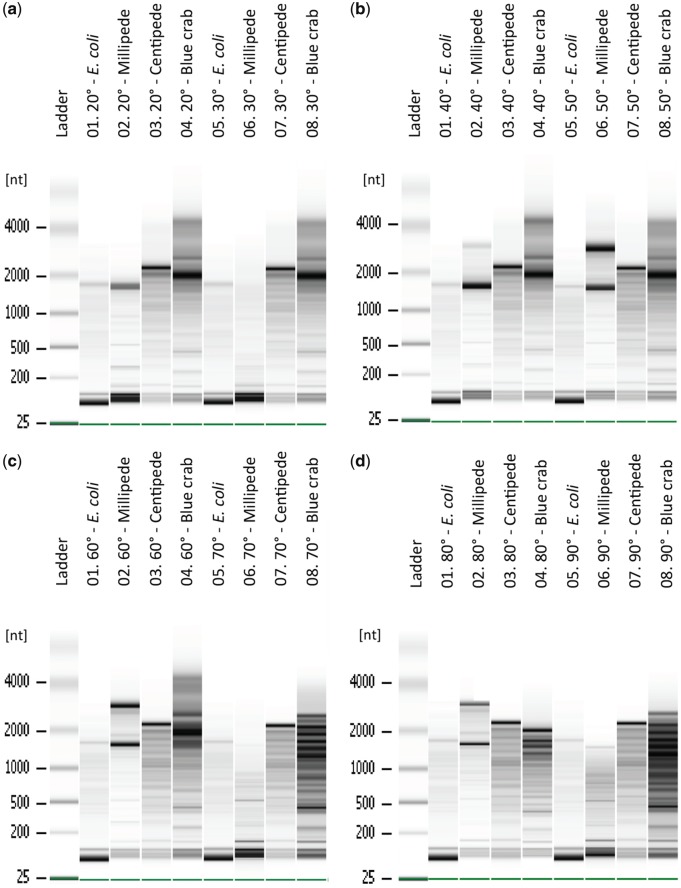
Temperature gradient (denaturing treatment) for three representative taxa and a positive control (See Table 1): *Escherichia coli* (Qubit Standard 2), Millipede (HBG5053), Centipede (HBG5058), and Blue crab (HBG5273). (a) 20–30°C, (b) 40–50°C, (c) 60–70°C, and (d) 80–90°C. Ladder corresponds to the picochip RNA size standard. These samples match those used in Supplementary Fig. S1.

The temperature gradient revealed that samples with the 28S “hidden break” (blue centipede and blue crab) had at least a portion of their 28S subunit already collapsed at 20°C (Fig. 2 and Supplementary Fig. S1). Blue crab RNA maintained some copies of its 28S subunit at ∼4000 bp until reaching 80°C. The positive bacterial control (*E. coli*) and the millipede (*A. monilicornis*) maintained 28S subunit integrity throughout the 20–90°C gradient, exhibiting a two-band profile reminiscent of the vertebrate rRNA model reference. The blue centipede (*H. marginata*) showed a single band throughout the entire gradient (Fig. 2 and Supplementary Fig. S1). Some lanes on the electropherograms (Fig. 2) showed RNA degradation not found on the agarose gel (Supplementary Fig. S1).

### High-throughput sequencing indicates high-quality RNA despite denaturation

**Figure 3:**
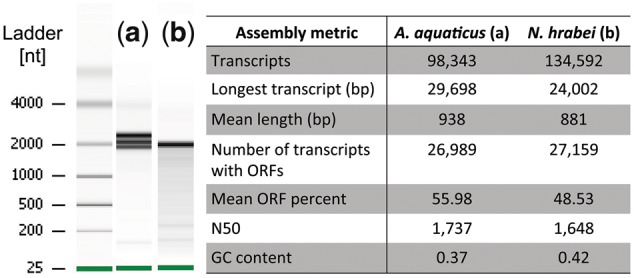
*De novo* transcriptome assembly statistics for the isopod *Asellus aquaticus* (**a**) and amphipod *Niphargus hrabei* (**b**), each exhibiting denatured RNA profiles indicative of 28S rRNA subunit collapse (left). Raw data were generated on an Illumina HiSeq4000 (BioProject PRJNA476149) using methods described in [37]. Transcriptomes were assembled using Trinity and assessed for quality and completeness using Transrate and BUSCO. Metrics include: total number of assembled transcripts, longest generated transcript (bp), mean length of all transcripts (bp), number of transcripts with an open reading frame (ORF), mean ORF percent, N50 statistic and overall GC content (see [37] for additional details).

Approximately 32 M paired-end reads were generated for the arthropods *A. aquaticus* and *N. hrabei*, exhibiting 28S denaturation (Fig. 3). *De novo* transcriptome assemblies yielded ∼98.3k and 134.5k transcripts/contigs, respectively (for more details, see [37]). Correspondingly, these assemblies had mean transcript lengths of 938 and 881 bp and N50 statistics of 1737 and 1648 bp (Fig. 3). BUSCO assessments, used to evaluate the completeness of the *de novo* transcriptomes, revealed 90.1% (C: 90.1%, F: 6.2%, M: 3.7%, *n*: 1066) and 90.5% (C: 90.5%, F: 5.3%, M: 4.2%, *n*: 1066), respectively, of the single-copy orthologs employed for benchmarking in Arthropoda were present, indicating fairly complete, high-quality assemblies.

## Discussion

### One size does not fit all: diversity of RNA banding profiles across arthropoda

There is a growing body of evidence suggesting that the current vertebrate RNA profile references are not reliable proxies to assess the quality of invertebrate RNA with seemingly “degraded” RNA profile patterns [21–23, 27, 28]. Our results suggest that 28S “collapse” or “denaturation” is a common occurrence across Arthropoda, but is likely a complex phenomenon resulting in diverse banding patterns, some of which are newly reported here. This renders model organism RNA profiles of limited use for various nonmodel taxa. Moreover, RINs could not be estimated for a majority of the samples exhibiting the denatured 28S rRNA subunits; paradoxically, RINs were only estimated for a few samples showing some level of “true” degradation [e.g., Fig. 1f (L01-03)]. Therefore, RIN estimation may be impossible or biased if algorithms do not account for denaturation and collapsed ribosomal subunits in a variety of invertebrates though manual bioanalyzer corrections (i.e., higher anomaly thresholds upon detection of the additional peak(s) or signal) can partially reduce bias [11]. In addition to deviations from the common rRNA two-banded profile, our results further show that rRNA 28S in invertebrates is smaller than that in vertebrates, 3900 bp versus 4400–5000 bp, though the 18S sizes appear to be within a 100 bp differential margin. Bacterial RNA profile sizes are even smaller (*E. coli* 2900 bp for 23S and 1700 bp for 16S; Fig. 1e). These estimated size differences will allow for a more accurate and prompt assessment of rRNA in nonmodel invertebrates following RNA extraction, including the presence of bacterial and human contamination.

The diversity of RNA profiles, ranging from one to five bands (Fig. 1 and Table 1), indicates that even though a one-band profile is the most frequently observed pattern among arthropods, individual lineages may vary. Some single bands within Insecta were also relatively thick, suggesting a possible artifact resulting from two high-quality overlapping bands of similar size. Denatured two-banded profiles, evident in several decapod crustaceans and insects [e.g., Fig.1a (L01), not to be mistaken with the banding profile of the vertebrate model reference], suggest that one of the two 28S fragments (α or β) is at least 100 bp longer than 18S and the other is very similar to 18S in length. The three-banded profile found among river isopods, mud and blue crabs, and *Daphnia* water fleas, suggests a significant translocation of the UAAU loop with 400 bp separating the two 28S fragments. Likewise, the possibility exists that a second UAAU region, or underlying secondary structure, is creating an additional cleavage point. A four-banded profile in tiger centipedes also implies that complex secondary structures within the loop region are creating additional cleavage points, possibly making them more accessible to enzymes (e.g., RNases). Alternatively, a secondary UAAU-rich loop may be present, resulting in three 28S fragments (α, β, and a novel γ) as tiger centipede RNA showed no signs of degradation (i.e., smears or a multitude of fragments). The third and fourth bands in tiger centipedes are also much smaller than bacterial rRNA subunits, rendering contamination from mitochondrial DNA or microorganisms unlikely. However, this four-banded pattern was not found in the blue centipede species (single band). This suggests that the four-banded pattern is not unanimous among Chilopoda. Similar to the RNA profiles observed in aphids [28, 42], millipedes (Diplopoda), the sister clade to centipedes, maintained the integrity of the 28S subunit, suggesting this four-banded pattern could be a genus or species-specific phenomenon.

The observed variation in RNA profiles and 28S denaturation found in this study is comparable to other systems. For example, a majority of mammals have a typical nondenatured two-banded vertebrate profile, but rodents in the genus *Ctenomys* have a denatured 28S subunit [17]. Thus, an important distinction exists between subunit collapse/denaturing and degradation. The generation of high-quality *de novo* transcriptome assemblies from RNAseq data for two arthropod species exhibiting denatured RNA profiles provides additional support for high-quality RNA despite 28S subunit collapse [37]. Assembly statistics for both species compare favorably with previously published RNAseq studies on *A. aquaticus* [43] and other amphipods [44], as well as arthropod species with nondenatured RNA profiles [45]. Denaturation appears to be an arthropod-wide phenomenon with some lineage-specific exceptions, whereas degradation remains constant due to the ephemeral nature of RNA [46]. For researchers working on organisms with unknown rRNA profiles, we therefore suggest documenting (replicated) rRNA banding patterns to make accurate assessments about RNA integrity and avoid falsely labeling 28S rRNA denaturing as “true” degradation.

### It's getting hot in here: the effect of temperature on 28S denaturation

A prior study by McCarthy *et al.* [21] examining rRNA degradation in a few arthropod lineages theorized 28S cleavage would occur at temperatures around 70°C as part of the Bioanalyzer protocol. Our temperature-gradient experiments showed that 28S denaturing can occur across a broad array of temperatures in arthropods, including room temperature (∼20°C). This can have important implications given human body temperatures are ∼37°C, and consequently excessive sample handling could contribute to unpredicted denaturing.

DNase treatments, which should be a standard practice in RNA extraction protocols, typically require heating samples to 37°C for enzyme activation. Therefore, 28S fragmentation will likely occur as part of the extraction protocol in all organisms with a UAAU-rich loop. Our findings also suggest that the large ribosomal subunit of some species may be resistant to denaturation as millipedes were able to maintain 28S integrity regardless of heat exposures to 90°C. This is possibly a result of a missing UAAU loop region similar to aphids (Hemiptera, Insecta; [28, 42]). Our results further show that if samples are exposed to temperatures beyond 70°C for enzymes requiring heat deactivation, RNA integrity can be severely impacted. However, it appears that DNases can be largely removed with a second round of alcohol precipitation; this allowed us to avoid high temperature incubations by employing an enzyme brand that does not require temperature deactivation. Thus, we recommend avoiding enzymes or denaturing treatments that require high temperatures in order to ensure RNA integrity.

### Denaturing gels versus electropherograms

Here, we show that both methods for evaluating RNA quality, denaturing gel electrophoresis and Bioanalyzer electropherograms, have their individual strengths while also being subjected to methodological artifacts. Though RIN values are unreliable in profiles where 28S is collapsed, electropherograms should still be used to assess RNA quality as they provide more accurate size estimates. Bleach agarose gels [36] can also be used for size estimation and to identify degradation or DNA contamination, in place of more toxic denaturing gels (e.g., Dimethyl sulfoxide (DMSO) and formaldehyde), to minimize time, effort, and costs. Furthermore, assessing RNA integrity via electrophoresis prior to diluting and running on a Bioanalyzer will allow researchers to more readily detect sources of degradation. For example, electropherograms for our gradient revealed degradation on a few lanes that were not initially present in the samples, indicating this occurred at some point during Bioanalyzer prep and/or transport. For these reasons, we recommend running samples on denaturing gels prior to or in conjunction with the bioanalyzer.

### Arthropod extraction techniques

Although comparing multiple RNA extraction methods are outside the scope of this study, we found one in particular that worked well for arthropods and downstream RNAseq studies. We ultimately selected solvent-based (phenol-chloroform; e.g., TRIZOL) over column-based methods (e.g., QIAGEN and NUCLEOSPIN), as it was cost-effective while also yielding higher concentrations of high-molecular weight nucleic acids as well as small and micro-RNAs. Column membranes sequestered too much material and often resulted in the isolation of ≪1 μg of total RNA per sample. Obtaining high RNA yields becomes particularly important when working with small-bodied organisms, a challenge faced by many invertebrate researchers. Lastly, solvent-based methods prove to be reliable across taxa and laboratories as the RNA profiles reported here are consistent with past arthropod RNA studies (e.g., one band in spiders, [47]; three bands in crabs [48]).

## Conclusions

Here we compared RNA profiles across Arthropoda from a diverse array of lineages including insects, crustaceans, arachnids, millipedes, and centipedes. We found that a majority of lineages displayed what would be incorrectly interpreted as a “degraded” RNA profile when compared with vertebrate references. Specifically, six distinct RNA banding patterns were revealed, providing insight into the high degree of variation that can be found across Arthropoda. However, 18S size (1900–2000 bp) remained constant among arthropods, making it a useful proxy for RNA integrity. Lastly, our study demonstrates that RNA is substantially labile and extraction protocol temperatures (>37°C) will denature 28S that contains the UAAU-rich loop region.

## Data accessibility 

Raw sequencing data are available on the National Center for Biotechnology Information (NCBI)’s Sequence Read Archive (SRA) database under Bioproject PRJNA476149. 

## Supplementary Material

Supplementary Figure S1

## References

[1] Chu Y, Corey DR. RNA sequencing: platform selection, experimental design, and data interpretation. Nucleic Acid Ther 2012;22:271–4. 22830413 10.1089/nat.2012.0367 PMC3426205

[2] Wang Z, Gerstein M, Snyder M. RNA-Seq: a revolutionary tool for transcriptomics. Nat Rev Genet 2009;10:57–63. 19015660 10.1038/nrg2484 PMC2949280

[3] Conesa A, Madrigal P, Tarazona S et al A survey of best practices for RNA-seq data analysis. Genome Biol 2016;17:13. 26813401 10.1186/s13059-016-0881-8 PMC4728800

[4] Hornett EA, Wheat CW. Quantitative RNA-Seq analysis in non-model species: assessing transcriptome assemblies as a scaffold and the utility of evolutionary divergent genomic reference species. BMC Genomics 2012;13:361. 22853326 10.1186/1471-2164-13-361 PMC3469347

[5] Achim K, Pettit JB, Saraiva LR et al High-throughput spatial mapping of single-cell RNA-seq data to tissue of origin. Nat Biotechnol 2015;33:503. 25867922 10.1038/nbt.3209

[6] Satija R, Farrell JA, Gennert D et al Spatial reconstruction of single-cell gene expression data. Nat Biotechnol 2015;33:495. 25867923 10.1038/nbt.3192 PMC4430369

[7] Houseley J, Tollervey D. The many pathways of RNA degradation. Cell 2009;136:763–76. 19239894 10.1016/j.cell.2009.01.019

[8] Florell SR, Coffin CM, Holden JA et al Preservation of RNA for functional genomic studies: a multidisciplinary tumor bank protocol. Mod Pathol 2001;14:116. 11235903 10.1038/modpathol.3880267

[9] Sambrook J, Russell DW. 2001 Molecular Cloning: A Laboratory Manual. Cold Spring Harbor, NY: CSH Laboratory Press.

[10] Imbeaud S, Graudens E, Boulanger V et al Towards standardization of RNA quality assessment using user-independent classifiers of microcapillary electrophoresis traces. Nucleic Acids Res 2005;33:e56. 15800207 10.1093/nar/gni054 PMC1072807

[11] Mueller O, Lightfoot S, Schroeder A. RNA integrity number (RIN)–standardization of RNA quality control. Agilent Application Note, Publication 2004;1:1–8.

[12] Fleige S, Pfaffl MW. RNA integrity and the effect on the real-time qRT-PCR performance. Mol Aspects Med 2006;27:126–39. 16469371 10.1016/j.mam.2005.12.003

[13] Fleige S, Walf V, Huch S et al Comparison of relative mRNA quantification models and the impact of RNA integrity in quantitative real-time RT-PCR. Biotechnol Lett 2006;28:1601–13. 16900335 10.1007/s10529-006-9127-2

[14] He S, Wurtzel O, Singh K et al Validation of two ribosomal RNA removal methods for microbial metatranscriptomics. Nat Methods 2010;7:807. 20852648 10.1038/nmeth.1507

[15] Opitz L, Salinas-Riester G, Grade M et al Impact of RNA degradation on gene expression profiling. BMC Med Genomics 2010;3:36. 20696062 10.1186/1755-8794-3-36 PMC2927474

[16] Jahn CE, Charkowski AO, Willis DK. Evaluation of isolation methods and RNA integrity for bacterial RNA quantitation. J Microbiol Methods 2008;75:318–24. 18674572 10.1016/j.mimet.2008.07.004

[17] Melen GJ, Pesce CG, Rossi MS et al Novel processing in a mammalian nuclear 28S pre‐rRNA: tissue‐specific elimination of an ‘intron’bearing a hidden break site. EMBO J 1999;18:3107–18. 10357822 10.1093/emboj/18.11.3107 PMC1171392

[18] Schroeder A, Mueller O, Stocker S et al The RIN: an RNA integrity number for assigning integrity values to RNA measurements. BMC Mol Biol 2006;7:3. 16448564 10.1186/1471-2199-7-3 PMC1413964

[19] Ødegaard F. How many species of arthropods? Erwin's estimate revised. Biol J Linnean Soc 2000;71:583–97.

[20] Potts SG, Biesmeijer JC, Kremen C et al Global pollinator declines: trends, impacts and drivers. Trends Ecol Evol 2010;25:345–53. 20188434 10.1016/j.tree.2010.01.007

[21] McCarthy SD, Dugon MM, Power AM. ‘Degraded’ RNA profiles in Arthropoda and beyond. PeerJ 2015;3:e1436. 26644977 10.7717/peerj.1436 PMC4671170

[22] Ishikawa HT, Newburgh RW. Studies of the thermal conversion of 28S RNA of *Galleria mellonella* (L.) to an 18 S product. J Mol Biol 1972;64:135–44. 5015395 10.1016/0022-2836(72)90325-7

[23] Fujiwara H, Ishikawa H. Molecular mechanism of introduction of the hidden break into the 28S rRNA of insects: implication based on structural studies. Nucl Acids Res 1986;14:6393–401. 3018670 10.1093/nar/14.16.6393 PMC311653

[24] Ware VC, Renkawitz R, Gerbi SA. rRNA processing: removal of only nineteen bases at the gap between 28S alpha and 28S beta rRNAs in Sciara coprophila. Nucleic Acids Res 1985;13:3581–97. 2989775 10.1093/nar/13.10.3581 PMC341260

[25] Sun S, Xie H, Sun Y et al Molecular characterization of gap region in 28S rRNA molecules in brine shrimp Artemia parthenogenetica and planarian *Dugesia japonica*. Biochemistry (Moscow) 2012;77:411–17. 22809161 10.1134/S000629791204013X

[26] Towle DW, Smith CM. Gene discovery in Carcinus maenas and Homarus americanus via expressed sequence tags. Integr Comp Biol 2006;46:912–8. 21672795 10.1093/icb/icl002

[27] Winnebeck EC, Millar CD, Warman GR. Why does insect RNA look degraded? J Insect Sci 2010;10:159. 21067419 10.1673/031.010.14119 PMC3016993

[28] Ogino K, Eda-Fujiwara H, Fujiwara H et al What causes the aphid 28S rRNA to lack the hidden break? J Mol Evol 1990;30:509–13. 2115929 10.1007/BF02101106

[29] Mallatt J, Chittenden KD. The GC content of LSU rRNA evolves across topological and functional regions of the ribosome in all three domains of life. Molecular phylogenetics and evolution 2014;72:17–30. 24394731 10.1016/j.ympev.2013.12.007

[30] Yakovchuk P, Protozanova E, Frank-Kamenetskii MD. Base-stacking and base-pairing contributions into thermal stability of the DNA double helix. Nucleic acids research 2006;34(2):564–74. 16449200 10.1093/nar/gkj454 PMC1360284

[31] Telford MJ, Bourlat SJ, Economou A et al The evolution of the Ecdysozoa. Philos Trans Royal Soc B 2008;363:1529–37. 10.1098/rstb.2007.2243 PMC2614232 18192181

[32] Oakley TH, Wolfe JM, Lindgren AR et al Phylotranscriptomics to bring the understudied into the fold: monophyletic ostracoda, fossil placement, and pancrustacean phylogeny. Mol Biol Evol 2013;30:215–33. 22977117 10.1093/molbev/mss216

[33] Rota-Stabelli O, Kayal E, Gleeson D, Daub J, Boore JL, Telford MJ, Pisani D, Blaxter M, Lavrov DV. Ecdysozoan mitogenomics: evidence for a common origin of the legged invertebrates, the Panarthropoda. Genome biology and evolution 2010;2:425–40. 20624745 10.1093/gbe/evq030 PMC2998192

[34] Wheeler WC, Whiting M, Wheeler QD et al The phylogeny of the extant hexapod orders. Cladistics 2001;17:113–69. 10.1111/j.1096-0031.2001.tb00115.x 34911238

[35] Bracken HD, Toon A, Felder DL et al The decapod tree of life: compiling the data and moving toward a consensus of decapod evolution. Arthropod Syst Phylo 2009;67:99–116.

[36] Aranda PS, LaJoie DM, Jorcyk CL. Bleach gel: a simple agarose gel for analyzing RNA quality. Electrophoresis 2012;33:366–9. 22222980 10.1002/elps.201100335 PMC3699176

[37] Pérez-Moreno JL, Balázs G, Bracken-Grissom HD. Transcriptomic insights into the loss of vision in Molnár János Cave’s crustaceans. Integr Comp Biol 2018;58:452–64. 29931265 10.1093/icb/icy071

[38] Andrews S. 2010 FastQC A Quality Control tool for High Throughput Sequence Data. accessed June 1, 2018 http://www.bioinformatics.babraham.ac.uk/projects/fastqc/

[39] Bolger AM, Lohse M, Usadel B. Trimmomatic: a flexible trimmer for Illumina sequence data. Bioinformatics 2014;30:2114–20. 24695404 10.1093/bioinformatics/btu170 PMC4103590

[40] Smith-Unna R, Boursnell C, Patro R, Hibberd JM, Kelly S. TransRate: reference-free quality assessment of de novo transcriptome assemblies. Genome research 2016;26:1134–1144. 27252236 10.1101/gr.196469.115 PMC4971766

[41] Simão FA, Waterhouse RM, Ioannidis P, Kriventseva EV, Zdobnov EM. BUSCO: assessing genome assembly and annotation completeness with single-copy orthologs. Bioinformatics 2015;31(19):3210–3212. 26059717 10.1093/bioinformatics/btv351

[42] Macharia RW, Ombura FL, Aroko EO. Insects’ RNA profiling reveals absence of ‘hidden break’ in 28S ribosomal RNA molecule of onion thrips, Thrips tabaci. J Nucleic Acids 2015;2015:1. 10.1155/2015/965294 PMC4342065 25767721

[43] Stahl BA, Gross JB, Speiser DI et al A transcriptomic analysis of cave, surface, and hybrid isopod crustaceans of the species *Asellus aquaticus*. PLoS One 2015;10:e0140484. 26462237 10.1371/journal.pone.0140484 PMC4604090

[44] Lan Y, Sun J, Tian R et al Molecular adaptation in the world’s deepest-living animal: insights from transcriptome sequencing of the hadal amphipod Hirondellea gigas. Mol Ecol 2017;26:3732–43. 28429829 10.1111/mec.14149

[45] Bansal R, Mian MAR, Mittapalli O et al RNA-Seq reveals a xenobiotic stress response in the soybean aphid, Aphis glycines, when fed aphid-resistant soybean. BMC Genomics 2014;15:1. 25399334 10.1186/1471-2164-15-972 PMC4289043

[46] Mattick JS. RNA regulation: a new genetics? Nature Reviews Genetics 2004;5(4):316. 10.1038/nrg1321 15131654

[47] Kono N, Nakamura H, Ito Y et al Evaluation of the impact of RNA preservation methods of spiders for *de novo* transcriptome assembly. Mol Ecol Resour 2016;16:662–72. 26561354 10.1111/1755-0998.12485

[48] Yednock BK, Sullivan TJ, Neigel JE. De novo assembly of a transcriptome from juvenile blue crabs (*Callinectes sapidus*) following exposure to surrogate Macondo crude oil. BMC Genomics 2015;16:521. 26162747 10.1186/s12864-015-1739-2 PMC4499174

