# Supplementary material for: RNA profile diversity across arthropoda: guidelines, methodological artifacts, and expected outcomes
Source: Biol Methods Protoc. 2018 Dec 15;3(1):bpy012. doi: 10.1093/biomethods/bpy012 (PMC6994094; doi:10.1093/biomethods/bpy012)
Supplement: Supplementary Figure S1 [file bpy012_figure_s1.pdf]

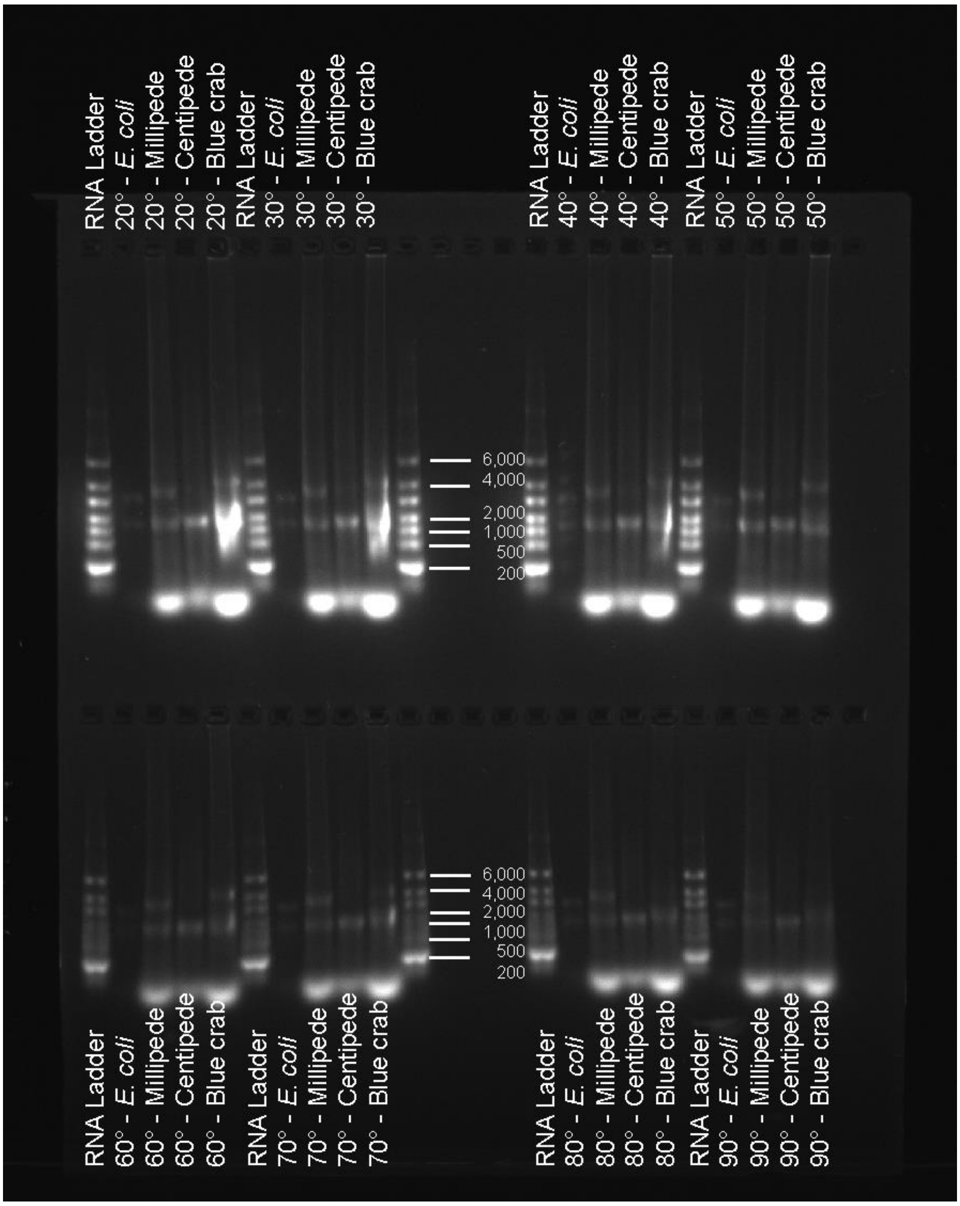

Figure S1- Temperature gradient (denaturing treatment) on a 1% TAE agarose bleach gel for three representative taxa and a positive control: *Escherichia coli* (Qubit Standard 2), Millipede (HBG5053), Centipede (HBG5058), and Blue crab (HBG5273). (A) 20 - 30ºC, (B) 40 - 50ºC, (C) 60 - 70ºC, and (D) 80 - 90ºC. RNA ladder fragment sizes (Thermofisher Riboruler) are given in base pairs (bp). These samples correspond to the electropherograms in Figure 2.
